# Auditory processing in children with normal and disordered speech

**DOI:** 10.1590/S1808-86942010000600009

**Published:** 2015-10-19

**Authors:** Victor Gandra Quintas, Tiago Mendonça Attoni, Márcia Keske-Soares, Carolina Lisbôa Mezzomo

**Affiliations:** 1 Master's degree student in Human Communication Disorders, UFSM. Speech therapist; 2 Master's degree student in Human Communication Disorders, UFSM. Speech therapist; 3 Doctoral degree in linguistics, PUC-RS. Professor in the graduate program Human Communication Disorders, UFSM; 4 Doctoral degree in linguistics, PUC-RS. Professor in the graduate program Human Communication Disorders, UFSM. Santa Maria Federal University (Universidade Federal de Santa Maria)

**Keywords:** child, speech disorders, auditory perception

## Abstract

Phonological speech disorders are characterized by abnormal development towards the adult target pattern; its etiology is unknown. It is thought the this condition results from auditory processing disorders involving the abilities required for human beings to understand what is heard.

**Aim:**

To investigate the relationship between auditory processing and the acquisition of disordered or normal speech, drawing comparisons between these profiles.

**Material and Method:**

A prospective, contemporary, cross-sectional study comprising a sample of 44 subjects aged 5 to 7 years; two groups were formed: a study group (SG) comprising children with disordered speech acquisition, and a control group (CG) consisting of children with normal speech acquisition. A simplified evaluation of auditory processing was undertaken: the PSI test in Portuguese; the speech-in-noise test; the binaural fusion test; the dichotic digit test; and the staggered spondaic word test (SSW).

**Results:**

There was a statistically significant difference between the two groups; the SG scored worse than the CG in all the tests. The PSI test only - with a 100% success rate - scored equally in both groups.

**Conclusion:**

Auditory processing may affect speech development.

## INTRODUCTION

Acquiring phonological abilities in most children takes place gradually and non-linearly from birth to age five years; there are individual variations, yielding an adult target system.^1^

Learning a language depends partly on learning which sounds are used and how they are organized. Most children carry out these actions effortlessly, and by age 5 years are able to adequately produce the sounds of the ambient language in the permitted sequences.^2^

Some children, however, do not acquire language satisfactorily and develop phonological disorders.^1^, ^2^

Phonological disorders may be defined as speech difficulties, characterized by inadequate language use according to age and regional variations, involving sound production, perception or organization errors. Disordered speech includes: replacements, omissions, insertions, transpositions, and/or distortions of language sounds.^3^, ^4^

Researchers have always been interested in studying phonological disorders; however, its causes remain unclear. It appears that several factors are involved in this disorder, such as sex, age, hearing loss, and the family context.^3^, ^4^, ^5^, ^6^

Oral language disorders, including phonological disorders, may be closely related with auditory processing disorders because hearing is the main entry path for language acquisition to be possible.^7^, ^8^, ^9^

The changes encompassed by the term “auditory processing” may be described as difficulties in processing specifically auditory information, in hearing or understanding speech, and in developing language abilities and learning.^10^

Auditory processing, therefore, includes the mechanisms and auditory system processes required for behavioral phenomena such as localization of sound, auditory discrimination and recognition, temporal resolution and ordering, auditory performance in competing sounds, and hearing performance in degraded auditory signals.^9^, ^11^

An auditory processing disorder has been defined as a loss in one or more of the abovementioned behaviors.^11^

There are not many papers to be studied in the literature on auditory processing and phonological disorders; this topic, however, can contribute significantly to the practice of phonoaudiology and related areas, since auditory processing appears to be linked to language acquisition and development.

The purpose of this article was to compare the development of auditory processing in normal speaking and phonologically disordered children.

## MATERIAL AND METHOD

This was a prospective contemporary cross-sectional study in which the phonological disorder is the independent variable and the results of auditory processing tests are the dependent variables.

The data were taken from clinical registries of children that participated in the research projects “Estudo dos desvios fonológicos: classificação e avaliação” (A study of phonological disorders: classification and assessment); it was approved by the institutional review board (no. 0093.0.243.000-09).

The data gathering period was from August to September 2009.

The inclusion criteria were as follows: authorization from parents and/or caretakers for participating in the study by signing a free informed consent form; child assent to participate; a diagnosis of phonological disorder (study group); male or female sex; age over 5 years 0 months (5:0).

The exclusion criteria were as follows: evident neurological, cognitive and/or psychological disorders; presence of hearing loss; disorders of the stomatognathic system that might affect speech; previous phonoaudiological therapy; difficulty to concentrate.

Children were selected from a screening program at a public health phonoaudiological clinic. The number of study subjects was based on the demand for phonoaudiological visits at our institution. The screening program evaluated 35 children, of which 22 met the sample inclusion criteria; the ages ranged from 5 years 0 months to 7 years 0 months (mean age - 6 years 3 months). There were 25 children initially selected from the screening program for the control group, of which 22 children of similar age to those in the control group were selected. An audiological assessment and phonoaudiological screening (language and stomatognathic system) were carried out as part of the sample selection process. Phonological and auditory processing assessments for data gathering are described below.

The phonological assessment was made using the Phonological Assessment for Children tool,^12^ which consists of five thematic drawings (bathroom, kitchen, front room, vehicles, and zoo) for gathering a sample of speech by naming and spontaneous speaking. Data were recorded and transcribed using the International Phonetic Alphabet, after which two expert speech therapists judged the transcriptions; inadequate transcriptions were discarded.

The tests - in Central Auditory Processing: Application Manual (Processamento Auditivo Central: Manual de Aplicação)^13^ - were: the simplified auditory processing evaluation, the binaural fusion test, the staggered spondaic word test, the dichotic digit test, and the speech-in-noise test. The standard age at which auditory abilities develop, and the published indications for tests according to age, were taken into account for each test.

The simplified auditory processing evaluation (for screening purposes) consists of three tasks; the first contains three sequences of non-verbal sounds, the second contains three sequences of verbal sounds, and the third consists of the sound localization task in five directions. This test is easily applied and requires only a quiet room and specific musical instruments - jingle bells, agogo, bells, and coconut shells. Three instrument sequences (nonverbal sounds) are used in the first step; subjects are asked to recall the sounds in the right order. The second step (verbal sounds) consists of three sequences of trisyllabic words that subjects are asked to recall in the right order. In these first two steps, subjects should provide correct answers for at least two of the three sequences in both tasks. The last step (localization of sound in five directions) consists of using a musical instrument (a bell) in five directions around the subjects (right side, left side, in front of the head, behind the head, and above the head); subjects must correctly confirm the lateral locations and at least two of the remaining directions.

The pediatric speech intelligibility (PSI) test may be applied ipsilaterally and contralaterally. Two conditions are tested: the MCI (two separate stimuli in the same ear), and the MCC (two separate stimuli, one for each ear). Verbal stimuli in the PSI test consist of ten phrases or words that should be identified by pointing at the corresponding figures. The competing message is a nursery rhyme. The signal-to-noise ratio is 0, -10 or -15 dB. Only the MCC condition was tested.

The speech-in-noise test consists of a list of words read ipsilaterally together with white noise. The signal-tonoise ratio may be 0 or +10 dB.

The binaural fusion test consists of a list of acoustically distorted monosyllabic words. This test assesses sensitivity at the brainstem. The received message (monosyllabic word) cannot be understood monotically; both ears need to integrate the sound.

The staggered spondaic word (SSW) test consists of a list of spoken words in free and competitive forms (right free, right competitive, left competitive, left free, and vice versa). This is the most useful test within the scientific community, as it may be applied in any population. Stimuli consist of 40 lists of four disyllabic words.

The dichotic digit test is a list of words (representing numbers) spoken simultaneously and dichotically. Subjects are asked to pay attention and repeat the words he or she hears. Next, subjects are asked to repeat only what was heard on one side, ignoring what was said to the contralateral ear. There are twelve lists divided into pairs, each one containing twenty words.

The tests were done in interference-free acoustic booths; a Fonix FA-12 two-channel clinical audiometer and TDH 39 earphones were used (ANSI S3.6/96: ANSI S343/92; ISO 389/91 calibration).

The Stata version 10.1 statistical software was used for ANOVA testing. The significance level was p < 0.05.

## RESULTS

The only auditory processing test in which all children attained 100% success was the PSI.

The scores in all other tests were higher in the control group compared to the study group; the latter evidenced disordered phonological acquisition.

Table 1 presents the test results - means and standard deviations. The p values for each subtest are also shown.

**Table 1:** Correct answers in the auditory processing tests for the study and control groups

Auditory processing tests		Study group	Control group	P
		Mean (SD)	Mean (SD)	
Screening	NVS	1.818 (1.006)	2.901 (0.294)	<0.001*
	VS	1.909 (1.191)	2.312 (0.476)	0.1424
	SL	4.045 (0.951)	4.909 (0.426)	<0.001*
	FA-RE	20.09 (5.701)	37.72 (2.711)	<0.001*
	FA-LE	20.40 (6.558)	38.00 (1.632)	<0.001*
Dichotic digits	AR-RE	19.72 (10.121)	39.22 (1.770)	<0.001*
	AR-LE	13.27 (8.597)	6.63 (3.073)	0.0707
	AL-LE	20.22 (8.106)	39.81 (0.588)	<0.001*
	AL-RE	12.31 (6.756)	1.77 (1.725)	<0.001*
Binaural fusion	RE	90.36 (7.371)	94.90 (4.308)	0.0165
	LE	87.88 (9.194)	96.54 (3.960)	<0.001*
Speech-in-noise	RE	94.77 (5.573)	96.54 (4.501)	0.2406
	LE	91.88 (7.352)	96.18 (4.856)	0.0251*
	NCR	4.545 (3.319)	18.90 (5.397)	<0.001*
SSW	CR	3.401 (2.481)	24.72 (5.666)	<0.001*
	CL	3.777 (2.910)	22.59 (4.305)	<0.001*
	NCL	3.909 (2.467)	17.54 (5.271)	<0.001*

Key: SD - standard deviation; NVS - non-verbal sequence; VS - verbal sequence; SL - sound localization; FA - free attention; AR; attention to the right; AL - attention to the left; RE - right ear; LE - left ear; NCR - non-competitive right; CR - competitive right; CL - competitive left; NCL - non-competitive left; p - Significance;

* - statistically significant.

There was no statistical significance between the control and study groups only in the verbal sounds sequence subtest of the simplified auditory processing evaluation. The same occurred in the right ear attention with left ear listening task in the dichotic digit test and the right ear listening test with speech-in-noise testing.

There were statistically significant differences between both groups in all other tests and tasks.

## DISCUSSION

According to the literature, the corpus callosum is fully mature only at age 7 years. This structure was still developing at the age range of our study subjects, but an idea of inter-hemispheric functions could already be gleaned.^14^ Auditory processing performance differences between the normal-speaking and the phonologically disordered groups were significant in this study.

The SSW and the dichotic digit tests yielded the highest mean differences among subjects in the sample, suggesting that children with disordered speech acquisition may have more difficulties in selective attention tests. The figure-background ability, wherein subjects understand a message in an environment with competing noise,^13^, ^15^ appears to be more affects in phonologically disordered subjects; this ability is evaluated in the tests described above.

Another relevant point is that better scores were attained in monaural tests compared to binaural tests in both groups; an example was the PSI test, in which all subjects reached maximum scores. This is possibly because messages are delivered ipsilaterally, where each ear operates individually.

A few papers have described a relation between auditory processing and phonology. One of them^16^ studied 91 subjects aged from 5 years 1 month to 6 years 11 months and found altered auditory processing in phonologically disordered public school students of a local town. The results of that study, based on the simplified auditory processing evaluation (screening), showed that the most frequent speech processes were decreased consonant clustering and substitution of liquid consonants. The authors concluded that a suggested change in auditory processing predominated in phonologically disordered children, and that a close relation between auditory processing abilities and speech function could not be denied.

Another more current study^17^ supported this putative relation. A survey was made of the registries of children aged at least 7 years. All children (100%) had at least one disordered auditory processing sub-profile, suggesting a close relation between auditory processing and phonological disorders. This underlines the importance of establishing whether auditory abilities in phonologically disordered children are compromised.

Our results concur with the hypothesis that children with disordered speech development may perform worse in auditory processing compared to normally speaking children.^16^, ^17^ The American Speech Hearing Language Association has suggested a cause-effect relation between language difficulties and auditory processing disorders, particularly in oral language understanding.^18^

The literature on this topic - auditory processing and phonological disorders - is sparse, but there are papers that relate auditory processing with language sub-areas; these papers cite phonology to some degree.^9^, ^19^, ^20^

Speech develops by perception for which hearing is the main path.^7^, ^8^, ^9^, ^10^, ^20^ This path may be misaligned if hearing mechanisms are abnormal, thereby compromising phonological acquisition and opening the road for phonological disorders. This is what we found in the present study; children in the study group performed worse than children in the control group.

We were thus able to confirm that auditory processing tests were abnormal in speech-disordered children.

## CONCLUSION

The data suggest a possible relation between auditory processing and phonological disorders, because children with speech acquisition disorders performed worse in auditory processing tests compared with normally-developing children.

Additional studies are always welcome to confirm these findings in larger samples, thereby supporting therapy for suppressing phonological disorders.
